# Two Portuguese Cochlear Implanted Dizygotic Twins: A Case Report

**DOI:** 10.1155/2012/623860

**Published:** 2012-08-23

**Authors:** Joana Rita Chora, Helena Simões-Teixeira, Tiago Daniel Matos, Jorge Humberto Martins, Marisa Alves, Raquel Ferreira, Luís Silva, Carlos Ribeiro, Graça Fialho, Helena Caria

**Affiliations:** ^1^Center for Biodiversity, Functional and Integrative Genomics, Faculty of Science, University of Lisbon, 1749-016 Lisbon, Portugal; ^2^ENT Department, Centro Hospitalar de Coimbra, Quinta dos Vales 3041-801 S. Martinho do Bispo, Coimbra, Portugal; ^3^School of Health, Polytechnic Institute of Setúbal, Largo Defensores da República, 2910-470 Setúbal, Portugal

## Abstract

Individual's hearing performance after cochlear implant (CI) is variable and depends on different factors such as etiology of deafness, age at implantation, and social/family hearing environment. Here we report the case of dizygotic twins, boy and girl, presenting with neurosensorial profound deafness prior CI (age of implantation = 3.5 years old). Both parents have severe/profound deafness, since childhood, and use sign language as primary mode of communication. Clinical and genetic characterization was performed, as well as the assessment of the auditory and oral (re)habilitation after CI, applying a battery of audiological, speech, and language tests. The twin girl and the father were homozygous for the c.35delG mutation in the GJB2 gene, while the twin boy and the mother were compound heterozygotes, both monoallelic for c.35delG and for the deletion del(*GJB6*-D13S1830) in the *GJB6* gene. The remaining hearing impaired relatives were c.35delG homozygotes. The genetic cause of deafness was thus identified in this family. Some noteworthy differences were observed regarding twins' auditory and oral performance after CI. Subsequent follow-up of these children allowed us to conclude that those differences were most likely due to the different environment in which the twins have been living than to their different *GJB2/GJB6* genotypes.

## 1. Introduction

After cochlear implantation (CI) the individual's hearing performance will vary according to their age at implantation [1], duration of implant use [2], level of residual hearing and mode of communication [3]. However, it has been documented that the contribution of these factors to speech perception after CI explains less than 50% of the variability observed [4], the remaining being related to other factors. One of these factors is thought to be the etiology of deafness [2, 5, 6], namely, connexin-associated deafness [7].

DFNB1 locus was the first nonsyndromic autosomal recessive deafness-related locus to be identified and is located on chromosome 13q11 [8]. It includes *GJB2* and *GJB6* genes, enconding the gap junction beta-2 protein (connexin 26) and the gap junction beta-6 protein (conexin 30), respectively. Mutations in the *GJB2* gene are the most frequent in nonsyndromic recessive deafness, accounting for up to 50% of the cases [9]. The c.35delG mutation, a deletion of a guanine in the *GJB2 *coding sequence, is the most common recessive deafness-causing mutation in Europe. Two large deletions, del(*GJB6*-D13S1830) and del(*GJB6*-D13S1854), identified in the *GJB6* gene, are often found in double heterozygosity with mutations in *GJB2*, which is thought to result in loss of function of the connexin cluster [10]. Most of these genotypes originate severe to profound congenital deafness, which are the cases recommended for cochlear implantation.

This study aimed at performing clinical and genetic characterization, as well as evaluating the oral (re)habilitation, of two nonidentical twins with cochlear implant.

## 2. Patient Presentation

**Figure 1:**
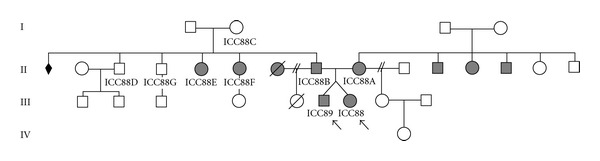
Family pedigree. Affected relatives are in full colour, the arrows signal the twins.

We report the case of dizygotic twins (a boy and a girl), aged 8 years, both with cochlear implants (CI). Both twins (Figure 1: ICC88, III : 7 and ICC89, III : 6) presented with neurosensorial profound hearing loss prior to implantation. Their parents (Figure 1: ICC88A, II : 9 and ICC88B, II : 8) are also severely to profoundly hearing impaired since childhood, use sign language as primary mode of communication, and have one unaffected daughter each, from a prior marriage, as well as several affected and unaffected relatives.

Table 1 presents the clinical history of the twins. It can be observed that both of them were diagnosed, started using hearing aids, and received their cochlear implant at the same time. Likewise, they use the same CI model with the same characteristics.

Written informed consent was obtained from all individuals and the study was approved by the Ethical Commission of the Hospital.

**Figure 2:**
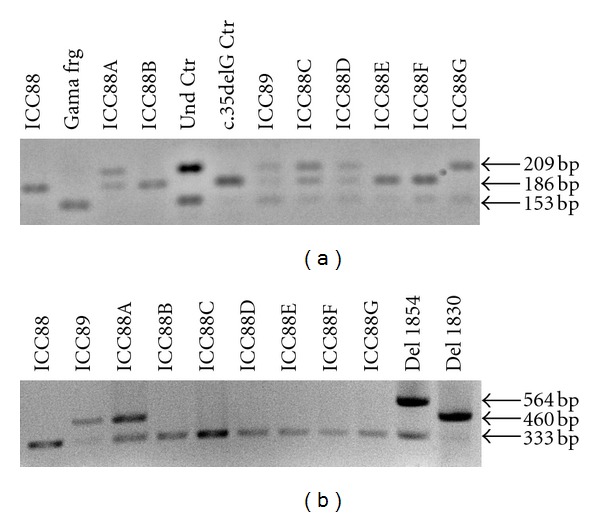
Electrophoresis results. (a) Enzymatic restriction with *Bsl*I for identification of c.35delG mutation according to Storm et al. [11]. Gama frg: internal restriction control (153bp band); Und Ctr: undigested restriction control (209 bp and 153 bp bands); c.35delG Ctr: c.35delG homozygous control (186 bp band). Homozygous mutated individuals present only the 186 bp band and heterozygous individuals present both the 209 bp and 186 bp bands. (b) PCR *multiplex* for identification of del(*GJB6*-D13S1830) and del(*GJB6*-D13S1854) according to del Castillo et al. [10]. Del 1854: heterozygous control for del(*GJB6*-D13S1854); Del 1830: heterozygous control for del(*GJB6*-D13S1830). The 333 bp band corresponds to nonmutated allele, the 460 bp band to del(*GJB6*-D13S1830) mutated allele and 564 bp band to del(*GJB6*-D13S1854) mutated allele.

DNA from the two twins and from affected and unaffected relatives was analysed, by sequencing and multiplex PCR, in respect to the presence of *GJB2 *coding mutations and *GJB6* deletions (del(*GJB6*-D13S1830) and del(*GJB6*-D13S1854)). Molecular analysis revealed that the twins present different genotypes: the girl (ICC088, III : 7) is homozygous for the c.35delG autosomal recessive mutation in *GJB2* while the boy (ICC089, III : 6) is a compound heterozygote for the c.35delG mutation and the *GJB6* large deletion del(*GJB6*-D13S1830) (Figure 2). Both genotypes are associated with severe congenital deafness phenotype. This genetic evaluation was included in a broader study involving DFNB1 genotype-phenotype correlation in Portuguese CI individuals [7], being the first time that del(*GJB6*-D13S1830) deletion was found in Portuguese deaf patients.

The different genotypes of the twins justified the genetic analysis of some of the twins' relatives (Figure 2). The father (ICC088B, II : 8) is a c.35delG homozygote, as the twin girl, and the mother (ICC088A, II : 9) is a c.35delG/del(*GJB6*-D13S1830) compound heterozygote like the twin boy. Accordingly, the affected relatives from the father's side (ICC088E, II : 5 and ICC088F, III : 6) are c.35delG homozygotes and the unaffected relatives either present one (ICC088C, I : 2 and ICC088D, II : 3) or none (ICC088G, II : 4) c.35delG alleles. None of them presented the *GJB6* deletion. On the mother's side no relative has been studied.

Assessment of the twins' global auditory and oral performance after cochlear implantation was carried out by applying a battery of audiological, speech, and language tests at the ENT Department from the Centro Hospitalar de Coimbra such as disyllabic words test discrimination score, monosyllable test, monosyllable phoneme test, number test, number phoneme test, sentences test, categories auditory performance (CAP), closed-set word perception test (with real objects, images, or written words), verbal articulation test (evaluates 19 consonants and 3 consonant groups, calculated as percentage of phonemes correctly produced), percentage of accurate vowels, vocal characteristics test (voice intensity, pitch, nasal resonance, intonation and breathing, phonation and articulation coordination), and speech intelligibility rating (SIR).

**Figure 3:**
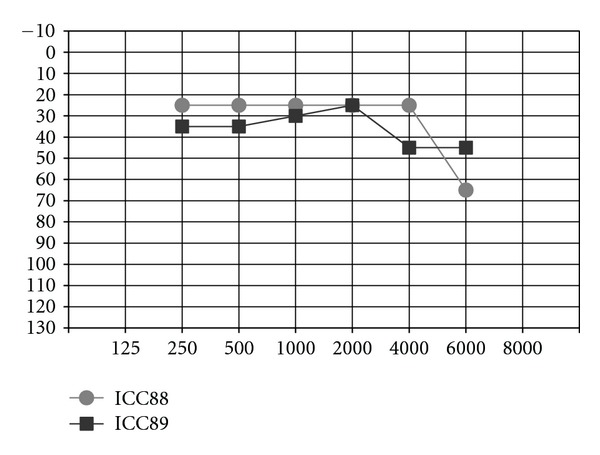
Pure-tone threshold levels of the twins after cochlear implantation (age 3, 5 years).

**Figure 4:**
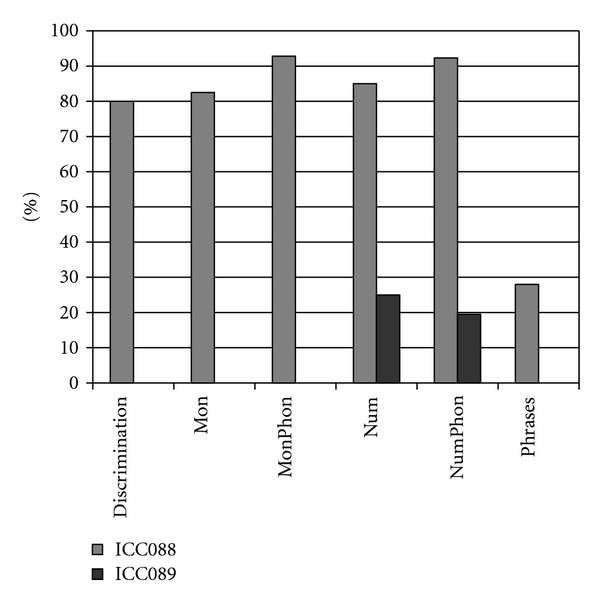
Twin's speech perception test's results (taken in 2009, 5 years of implant use). Disyllabic words test discrimination score, Monosyllable test, Monosyllable phoneme test, Number test, Number phoneme test, and Phrases: sentences test.

When observing the twins' audiograms at 3.5 years old, just after CI, (Figure 3), the pure tone thresholds for most frequencies were very similar. Thus, it would be reasonable to assume their auditory and oral performance scores would also be similar. However the speech perception tests performed after 5 years of CI use (Figure 4), show that the twin girl (ICC88, III : 7) had an overall good performance throughout the tests while the twin boy (ICC89, III : 6) got poorer results, had difficulties regarding some of the age appropriate tests that are part of the standard evaluation, and was not even able to respond to some of the tests.

**Figure 5:**
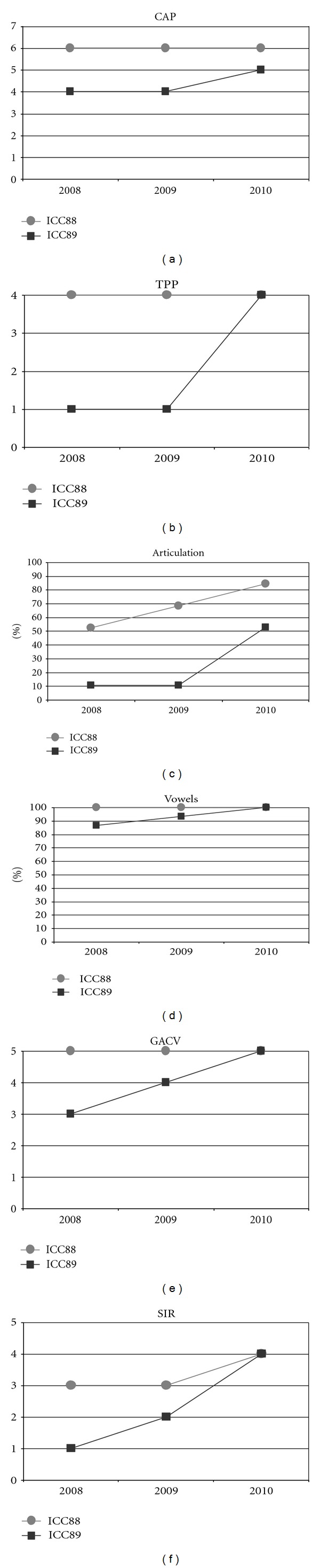
Twins' audiological test results in the last three years. (a) CAP: Categories Auditory Performance; (b) TPP: closed-set word perception test; (c) Articulation: verbal articulation test; (d) Vowels: percentage of accurate vowels; (e) GACV: Vocal characteristics test (voice intensity, pitch, nasal resonance, intonation and breathing, etc.); (f) SIR: Speech Intelligibility Ratting.

Could the differences in auditory and oral outcomes on post-CI rehabilitation be related to the different DFNB1 genotypes? Further analysis of the family history showed that other factors could be playing an important role and contributing for the differences observed. The twins had not lived together in the same hearing environment as previously assumed. On the contrary, each of them came from a different social background. The twin girl has lived since she was 6 months-old with their hearing aunt, and uncle (II : 1 and II : 2) and has been educated in a regular school with special education and speech therapy while the twin boy lived until 7 years-old with the hearing impaired parents, who only used sign language as mode of communication, thus in a poor auditory stimulating environment, only having contact with other deaf children, and with some household problems. In 2009, through legal decision, he moved in with his sister, aunt and uncle (II : 1 and II : 2) and integrated regular school with special education and speech therapy. The audiological tests from the last three years revealed the twin boy's favourable evolution since this social alteration (Figure 5). The positive effect of the auditory and oral stimulation observed in the twin since he moved to his aunt highlights the importance of the auditory stimulating environment in the success of the post-CI rehabilitation.

## 3. Conclusion

The nonidentical twins here analysed, aged 8 years-old presented different genotypes, c.[35delG]+[35delG] and [c.35delG]+[del(*GJB6*-D13S1830)], and different speech perception results after CI. The twin boy presented weaker verbal outcomes and worse level of residual hearing before he was implanted (absence of AEP's). However, both twins' audiograms were very alike after CI, revealing that their hearing thresholds, unlike their auditory and oral performance, were similar.

As such, the observed differences in the oral performance are most likely due to the different social context in which the twins have been living and not to their different *GJB2/GJB6* genotype. The oral outcome of the twin boy improved from the moment he started living in a hearing stimulating environment, which is a strong evidence of this factor's importance in the success of the oral rehabilitation after CI, namely, in DFNB1-associated hearing loss cases.

The remaining twins' affected relatives studied carried the c.35delG mutation in homozigosity. Molecular diagnosis and genetic counselling is thus very important to a family such as this one, namely, to the twins' unaffected half-sister still alive.

## Figures and Tables

**Table 1:** Clinical history of the twins.

	ICC088 (III : 7)	ICC089 (III : 6)
Gender	Female	Male
Deafness etiology	Congenital	Congenital
Suspicion of deafness	18 months	18 months
Diagnosis	24 months	24 months
Clinical history	Normal pregnancy No associated pathologies No previous surgical procedures 2 episodes of ear infections (otitis) prior implantation (without suppuration) No allergies Vaccination plan up to date	Normal pregnancy No associated pathologies No previous surgical procedures No allergies Vaccination plan up to date
Audiogram	Neurosensorial bilateral profound deafness, stage 3	Neurosensorial bilateral profound deafness, stage 3
Placement of hearing aids	24 months	24 months
Gain with hearing aids	Very poor adaptation No language development	Good adaptation Functional gain up to 1000 Hz No language development
Auditory evoked potentials (AEP)	Wave V present above 90 dB nHL on the right and above 100 dB nHL on the left	Absence of AEPs until 110 dB nHL bilaterally
Computerized axial tomography scan (CAT scan)	Morphologically normal and permeable cochlea Normal inner ear canals	Morphologically normal and permeable cochlea Normal inner ear canals
Magnetic resonance imaging (MRI)	No morphological alterations No congenital malformations No cochlear nerve atrophy Normal cochleas	No morphological alterations No congenital malformations No cochlear nerve atrophy Normal cochleas
Age at implantation	42 months	42 months
Duration of CI use	56 months	56 months
Age at audiological tests and present study	8 years	8 years
CI model	CI24R CA Advance	CI24R CA Advance
Speech processor	SPRINT	SPRINT
Stimulation mode	MP1+2	MP1+2
Stimulation strategy	ACE	ACE
Implanted ear	Right	Right
